# Special Issue “New Insights into Photosystem I”

**DOI:** 10.3390/ijms26178185

**Published:** 2025-08-23

**Authors:** Stefano Santabarbara, Gary Hastings

**Affiliations:** 1 Photosynthesis Research Unit, Consiglio Nazionale delle Ricerche, Via A. Corti 12, 20133 Milan, Italy; 2 Department of Physics and Astronomy, Georgia State University, Atlanta, GA 30303, USA

Photosystem I (PSI) is a central component in oxygenic photosynthesis. It catalyzes the light-driven oxidation of plastocyanin (or cytochrome *c_6_*) and the reduction of ferredoxin (or flavodoxin). Similar to other light-dependent oxidoreductases in photosynthesis, PSI features a highly complex architecture, comprising 12 or more protein subunits that collectively bind over 200 cofactors.

PSI is structurally and functionally intricate and has been the subject of investigations using a wide array of biological, spectroscopic, and structural techniques (several of which are highlighted in this Special Issue). These efforts have yielded advanced insights into PSI’s functionality, although key mechanistic aspects—such as photochemical charge separation—remain elusive and are active areas of investigation.

Structurally, PSI comprises two principal units: a conserved core complex and a variable external light-harvesting system. The core, housing the photocatalytic reaction center and electron transfer cofactors, is largely preserved across taxa—reflecting its essential role in synchronizing with other photosynthetic supercomplexes, such as Photosystem II, Cytochrome *b6f*, and ATP synthase. In contrast, the external antenna system exhibits substantial diversity in protein and pigment composition, shaped by environmental factors such as light spectrum, intensity, and fluctuation. This variability enables photosynthetic organisms to optimize photon capture while maintaining high photochemical efficiency. Understanding the principles of light harvesting optimization, in conjunction with the attainment of high photochemical efficiency, is an area of active research. The current set of manuscripts in this Special Issue represents a snapshot of these research activities.

This Special Issue features the following collection of original and review articles addressing the structural and functional diversity of PSI:

Nelson [1] and Tian & Chen [2] utilize the rapidly evolving capabilities of cryo-electron microscopy to explore light-harvesting architecture in algae and cyanobacteria.

The unique long-wavelength chlorophyll absorption features of PSI (>700 nm), particularly abundant and variable in cyanobacteria, are the focus of work by Zazubovich & Jankowiak [3], who investigate site energies via high-resolution site-selected spectroscopy. van Stokkum et al. [4] examine the dynamics of excited states and photochemical conversion across multiple cyanobacterial species.

Eden & Renger [5] present computational methods to model chromophore–chromophore and chromophore–protein interactions—critical for both light harvesting and electron transfer. Their parameterized approaches offer substantial reductions in computational cost compared to traditional low-level methods.

Electron transfer mechanisms are further explored in studies by Kirpich et al. [6], Luo et al. [7], Bindra et al. [8], and Santabarbara & Casazza [9]. Kirpich et al. and Luo et al. examine the cofactors at eC2 (A_–1_) and eC3 (A_0_), respectively, assessing how protein coordination modifications—introduced via site-specific mutations in the PsaA and PsaB proteins—affects reactivity.

Bindra et al. employ high-frequency electron paramagnetic resonance (EPR) spectroscopy to investigate phylloquinone radical pairs (A_1A_/A_1B_) and their species-dependent energetics, which influence reduction of the downstream iron–sulfur cluster F_X_. Despite variations, bidirectional electron transfer remains functional across all systems studied.

Santabarbara & Casazza analyze the thermodynamics and kinetics of electron transfer involving the iron–sulfur clusters F_X_, F_A_, and F_B_, providing predictive models that compensate for limited direct measurements.

Finally, Kehler et al. [10] explore the integration of PSI into biohybrid photovoltaic devices. They highlight PSI’s near-unity quantum efficiency and structural robustness as promising traits for sustainable energy applications.
